# Supplementary material for: Synthesis of Cyclic Peptides in SPPS with Npb-OH Photolabile Protecting Group
Source: Molecules. 2022 Mar 29;27(7):2231. doi: 10.3390/molecules27072231 (PMC9000773; doi:10.3390/molecules27072231)
Supplement: Supplementary file 1 [file molecules-27-02231-s001.zip › molecules-1617536-supplementary.pdf]

Supporting Information

Synthesis of cyclic peptides in SPPS with Npb-OH photolabile protecting group

Tingting Chen, Gang Wang*, Lin Tang, Hongpeng Yang, Jing Xu, Xiaoxue Wen, Yunbo Sun, Shuchen Liu, Tao Peng*, Shouguo Zhang*, and Lin Wang*

Institute of Radiation Medicine, Beijing 100850, China

1. Reaction procedures

*1.1. Synthesis of 1-(2-Nitrophenyl) propan-2-one (**1**)*

A solution of ethylacetoacetate (6.51 g, 50 mmoL) in 10 mL of DMF was slowly added to a suspension of 60% sodium hydride (3.01 g, 75 mmoL) in 60 mL of DMF in ice-bath conditions for 2 h (light yellow). Then, o-fluoronitrobenzene (7.06 g, 50 mmoL) was added, and the reaction mixture was stirred at r.t for 10 h. The reaction mixture was poured onto ice water, adjusted to a weak acidity with 2 M HCl (pH 5~6), and extracted with EtOAc (3 × 30 mL). The extracts were washed with saturated brine and dried over Mg_2SO_4. The solution was filtered, the filtrate was concentrated to dryness under vacuum, and the residue was purified to yield 10.30 g (82.0%) of β-keto ester as a yellow oil with flash column chromatography (petroleum ether: EtOAc = 20:1)[1]. The β-keto ester was dissolved into 30 mL of DMSO and 5 mL of brine, which were heated to 80 ℃ for 10 h. Next, 10 mL of water was added to the cooled mixture, followed by extraction with EtOAc (3 × 30 mL). The combined organic layer was washed with brine, dried, filtered, and dried under vacuum. The residue was purified with flash column chromatography (PE: EtOAc = 10:1) to yield 6.37 g (84.7% yield) of a light yellow/brown oil product[2].

*1.2. Synthesis of 3,3-dimethyl-1-(2-nitrophenyl)butan-2-ol (**7**)*

A suspension of potassium tert-butoxide (0.6 g, 5 mmoL) in 10 mL DMSO and 10 mL Tert-butanol was added to a solution of 2-nitrotoluene (6.85 g, 50 mmoL) and pivalaldehyde(4.3 g, 50 mmoL) in 30 mL DMSO (red wine).,which were stirred over night at r.t. The reaction mixture was evaporated under vacuum, and then 400 mL 2M HCl was added, the mixture was extracted with DCM (3 × 30 mL). The organic portion was washed with saturated brine, dried over Mg_2SO_4, and filterated. The filtrate was concentrated to dryness under vacuum, and the residue was purified by flash column chromatography to obtain 5.20 g as yellow prismatic crystal, yield, 46.6%; m.p. = 64-66℃[3]. ^{1}H NMR (400 MHz, DMSO-d_6) δ 7.86 (d, J = 8.1 Hz, 1H), 7.61 (t, J = 7.5 Hz, 1H), 7.50 (d, J = 8.1 Hz, 1H), 7.45 (t, J = 7.5 Hz, 1H), 4.57 (d, J = 6.4 Hz, 1H), 3.16 (dd, J = 10.8, 6.4 Hz, 1H), 3.05 (d, J = 13.4 Hz, 1H), 2.74 (dd, J = 13.3, 10.7 Hz, 1H), 0.89 (s, 9H). ^{13}C NMR (150 MHz, DMSO-d_6) δ 150.7, 135.7, 133.9, 132.8, 127.5, 124.3, 78.6, 35.7, 34.4, 26.3, 26.3, 26.3. HRMS (ESI-TOF) caled for $C_{12}H_{17}NO_3$ [M+Na]$^+$ 246.1106, Found 246.1106. The structure of derivative **7** was confirmed by the ^{1}H- and ^{13}C-NMR spectra (Figure S6).

*1.3. Synthesis of 2-(2-nitrophenyl)-1-phenylethan-ol (**6**)*

Operation as above compound (**7**), the aldehyde was convert to benzaldehyde. Yield, 56.8%; yellow solid; m.p.= 72-74℃ [4].

1.4. General oxidation procedure

1.4.1. Synthesis of 2-(2-nitrophenyl)-1-phenylethan-1-one (**8**)

2-(2-nitrophenyl)-1-phenylethan-1-ol (**6**) were dissolved in 50 mL DCM, PCC (5.80g, 27 mmoL) was poured onto. The mixture was stirred in the dark for 6 h. The reaction solution was filtered through a short silica gel column, eluted with PE: EA = 10:1, and the filtrate was collected and evaporated to give 3.68 g 2-(2-nitrophenyl)-1-phenylethan-1-one (**8**) as orange oil; yield, 88.0%[5].

1.6.2. Synthesis of 3,3-dimethyl-1-(2-nitrophenyl)butan-2-one (**9**)

For the new compounds 3,3-dimethyl-1-(2-nitrophenyl)butan-2-one (**9**), it has been characterized as follows. The reaction was performed as described in the procedure for compound **8**, starting from 3,3-dimethyl-1-(2-nitrophenyl)butan-2-ol (**7**). Column chromatography eluent, PE:EA = 10:1. 4.60 g as yellow oil, yield, 79.6% [6]. ^{1}H NMR (400 MHz, DMSO-d_6) δ 8.07 (d, J = 8.1 Hz, 1H), 7.69 (t, J = 7.5 Hz, 1H), 7.54 (t, J = 8.0 Hz, 1H), 7.47 (d, J = 7.6 Hz, 1H),4.40 (s, 2H), 1.18 (s, 9H). ^{13}C NMR (150 MHz, DMSO-d_6) δ 211.4, 149.5, 134.2, 134.0, 131.5, 128.7, 125.0, 44.0, 42.4, 26.8, 26.8, 26.8. HRMS (ESI-TOF) caled for $C_{12}H_{15}NO_3$ $[M+Na]^+$ 244.0950, Found 244.0945. The structure of derivative **9** was confirmed by the ^{1}H- and ^{13}C-NMR spectra (Figure S7).

1.5. General methylation procedure

The solution of THF and 1-(2-nitrophenyl) propan-2-one **1** (0.72 g, 4 mmoL) was cooled while stirring in an ice bath, and then, MeI (0.68 g, 4.8 mmoL) and 60% NaH (0.21 g, 5.2 mmoL) were added dropwise (violet). After stirring for 30 min and then at r.t for 6 h, the mixture was evaporated and the residue was dissolved in 2 M HCl (10 mL) and extracted with DCM (3 × 10 mL). The organic portion was washed with an $Na_2S_2O_3$ solution (3 × 10 mL) and saturated brine (3 × 10 mL), and then, it was dried over $MgSO_4$. The solution was filtered, the filtrate was concentrated to dryness under vacuum, and the residue was purified with flash column chromatography (column chromatography eluent; petroleum ether: EtOAc = 20:1) to afford product **2**. The compounds **10**, **11, 14** and **15** were characterized as follow.

3-(2-nitrophenyl)butan-2-one (**2**). The yield of this reaction was 0.39 g, 50.6%. Light yellow oil. It was identified by comparing its physical data with the literature data [7].

2-(2-nitrophenyl)-1-phenylpropan-1-one (**10**). The synthesis process as above compound (**2**), the starting material was convert to 2-(2-nitrophenyl)-1-phenylethan-1-one (**8**), base was transformed to KTB. The yield of this reaction was 1.90 g, 31.9%. Column chromatography eluent, PE:EA = 10:1. Yellow oil. It was identified by comparing its physical data with the literature data[7].

2,2-dimethyl-4-(2-nitrophenyl)pentan-3-one (**11**). The synthesis process as above compound (**2**), the starting material was convert to 3,3-dimethyl-1-(2-nitrophenyl)butan-2-one (**9**), base was transformed to KTB. 2.11 g, 48.4% yield. Column chromatography eluent, PE: EA = 10:1. Light yellow oil. ^{1}H NMR (400 MHz, DMSO-d_6) δ 7.93 (d, J = 8.1 Hz, 1H), 7.70 (t, J = 7.7 Hz, 1H), 7.54 (t, J = 7.7 Hz, 1H), 7.45 (d, J = 7.9 Hz, 1H), 4.68 (q, J = 6.8 Hz, 1H), 1.36 (d, J = 6.8 Hz, 3H), 1.02 (s, 9H). ^{13}C NMR (150 MHz, DMSO-d_6) δ 214.6, 149.4, 134.6, 133.7, 129.4, 128.8, 124.9, 44.8, 40.9, 26.9, 26.9,

26.9, 20.0. HRMS (ESI-TOF) caled for $C_{13}H_{17}NO_3$ $[M+H]^+$ 236.1287, Found: 236.1280. The structure of derivative **11** was confirmed by the 1H- and 13C-NMR spectra (Figure S8).

2-(2-nitrophenyl)cyclopentan-1-one (**14**). The synthesis process as above compound (**2**), the starting material was convert to *o*-fluoronitrobenzene and 1-(cyclohex-1-en-1-yl)pyrrolidine. The yield of this reaction was 1.08 g, 17.5% yield. Column chromatography eluent, PE: EA = 20:1. Light yellow oil. It was identified by comparing its physical data with the literature data[7].

2-(2-nitrophenyl)cyclohexan-1-one (**15**). The synthesis process as above compound (**2**), the starting material was convert to *o*-fluoronitrobenzene and 1-(cyclopent-1-en-1-yl)pyrrolidine. The yield of this reaction was 2.88 g, 26.3% yield. Column chromatography eluent, PE: EA = 20:1. Yellow oil. It was identified by comparing its physical data with the literature data[7].

1.6. Synthesis of 3-(2-nitrophenyl)but-3-en-2-one(3)

In a 100 mL eggplant flask with 1- (2-nitrophenyl) propyl - 2-one (4.32 g, 24 mmoL), 30 mL methanol was added and stirred at room temperature. The reaction should be protected from light throughout the reaction. Paraformaldehyde (0.86 g, 28.8 mmol) was added to the reaction. Piperidine (0.20 g, 2.4 mmol) and glacial acetic acid (0.14 g, 2.4 mmol) were added dropwise to the reaction, heated to reflux at 60 °C, and the reaction progress was monitored by TLC. After 4 h, it was cooled to room temperature, diluted with 20 mL of water, extracted with ethyl acetate (3×20 mL), the organic layers were combined, washed with saturated sodium chloride, dried over anhydrous magnesium sulfate, filtered, and evaporated to dryness to obtain brown 4.11 g of brown oily crude product was purified by silica gel column chromatography (petroleum ether: ethyl acetate=10: 1) to obtain 3.40 g of brown oily pure product, yield, 69.2%[8].

Reference

1. Ruhland, B. and L.G. Synthesis of 1-Hydroxy-2H,5H-Dihydroisoxazolo[5,4-c]Quinoline. A Novel Heterocyclic Ring System. *J. Heterocyclic Chem* **1989**, *26*, 469–471, doi:https://doi.org/10.1002/jhet.5570260240.
2. Nykaza, T. v.; Li, G.; Yang, J.; Luzung, M.R.; Radosevich, A.T. P III /P V =O Catalyzed Cascade Synthesis of N-Functionalized Azaheterocycles. *Angewandte Chemie* **2020**, *132*, 4535–4540, doi:10.1002/ange.201914851.
3. Alexander Stranub, W.J.N.G.L.N.L.O. Method for Producing Alkenylnitrobenzene Derivatives Unbranched in the 1'-Positon 2010, A1.
4. Gao, D.M.; Ma, W.L.; Li, T.R.; Huang, L.Z.; Du, Z.T. An Improved Synthesis of 1,2-Diarylethanols under Conventional Heating and Ultrasound Irradiation. *Molecules* **2012**, *17*, 10708–10715, doi:10.3390/molecules170910708.
5. Coffman, K.C.; Palazzo, T.A.; Hartley, T.P.; Fettinger, J.C.; Tantillo, D.J.; Kurth, M.J. Heterocycle-Heterocycle Strategies: (2-Nitrophenyl)Isoxazole Precursors to 4-Aminoquinolines, 1 H-Indoles, and Quinolin-4(1 H)-Ones. *Organic Letters* **2013**, *15*, 2062–2065, doi:10.1021/ol400787y.
6. Gall1, C. Evidence for a Non-Chain SRN1 Reaction Occurring on a Nitroarylhalide. *Tetrahedron* **1988**, *44*, 5205–5208, doi:10.1016/S0040-4020(01)86027-0.

7. Bühler, S.; Lagoja, I.; Giegrich, H.; Stengele, K.P.; Pfleiderer, W. New Types of Very Efficient Photolabile Protecting Groups Based upon the [2-(2-Nitrophenyl)Propoxy]Carbonyl (NPPOC) Moiety. *Helvetica Chimica Acta* **2004**, *87*, 620–659, doi:10.1002/hlca.200490060.
8. Okuro, K.; Gurnham, J.; Alper, H. Ionic Diamine Rhodium Complex Catalyzed Reductive N-Heterocyclization of 2-Nitrovinylarenes. *Journal of Organic Chemistry* **2011**, *76*, 4715–4720, doi:10.1021/jo200320k.

Table of contents

Figure S1. HPLC chromatograms and identification of cyclic peptide Ⅰ.

Figure S2. HPLC chromatograms and identification of cyclic peptide Ⅱ.

Figure S3. HPLC chromatograms and identification of cyclic peptide Ⅲ.

Figure S4. ^{1}H NMR and ^{13}C NMR spectra of compounds **4**.

Figure S5. ^{1}H NMR and ^{13}C NMR spectra of compounds **5**.

Figure S6. ^{1}H NMR and ^{13}C NMR spectra of compounds **7**.

Figure S7. ^{1}H NMR and ^{13}C NMR spectra of compounds **9**.

Figure S8. ^{1}H NMR and ^{13}C NMR spectra of compounds **11**.

Figure S9. ^{1}H NMR and ^{13}C NMR spectra of compounds **12**.

Figure S10. ^{1}H NMR and ^{13}C NMR spectra of compounds **13**.

Figure S11. ^{1}H NMR and ^{13}C NMR spectra of compounds **16**.

Figure S12. ^{1}H NMR and ^{13}C NMR spectra of compounds **17**.

Figure S13. ^{1}H NMR and ^{13}C NMR spectra of compounds **4b**.

Figure S14. ^{1}H NMR and ^{13}C NMR spectra of compounds **4c**.

Figure S15. ^{1}H NMR and ^{13}C NMR spectra of compounds **4d**.

Figure S16. ^{1}H NMR and ^{13}C NMR spectra of compounds **4e**.

Figure S17. ^{1}H NMR and ^{13}C NMR spectra of compounds **4f**.

Figure S18. ^{1}H NMR and ^{13}C NMR spectra of compounds **4g**.

Figure S19. ^{1}H NMR and ^{13}C NMR spectra of compounds **4h**.

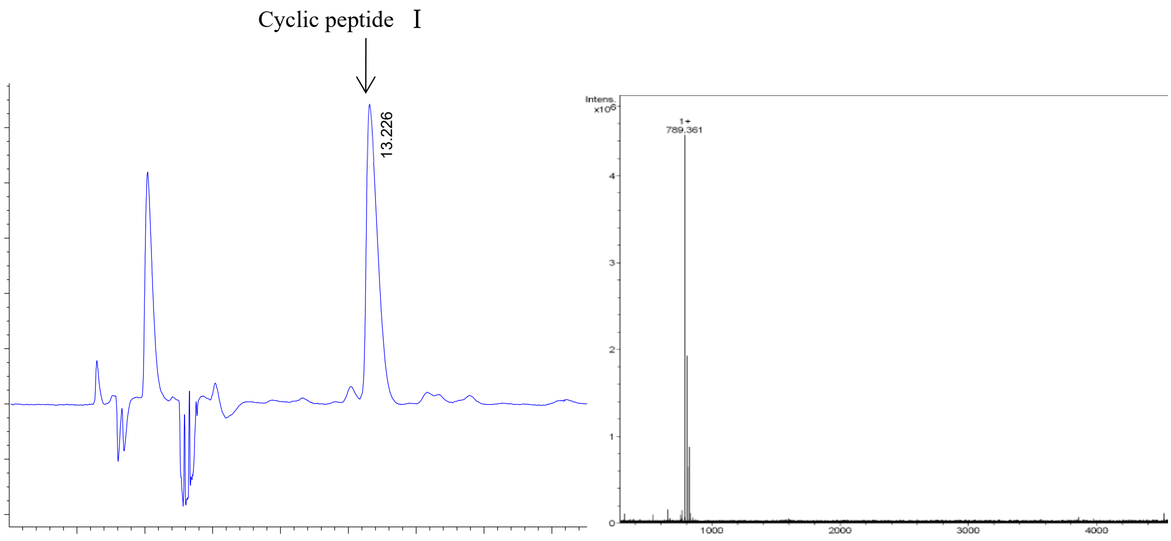

Figure S1. HPLC chromatograms and identification of cyclic peptide Ⅰ. HPLC trace (left) with 8 % CH_3CN–H_2O (0.1% TFA) as eluent system.

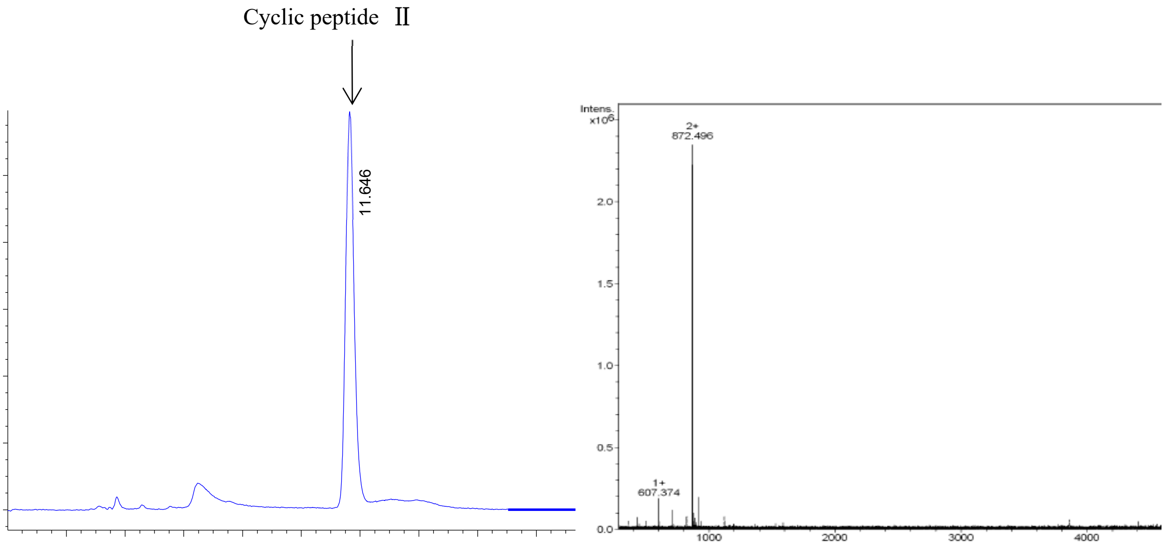

Figure S2. HPLC chromatograms and identification of cyclic peptide Ⅱ. HPLC trace (left) with 30 % CH_3CN–H_2O (0.1% TFA) as eluent system.

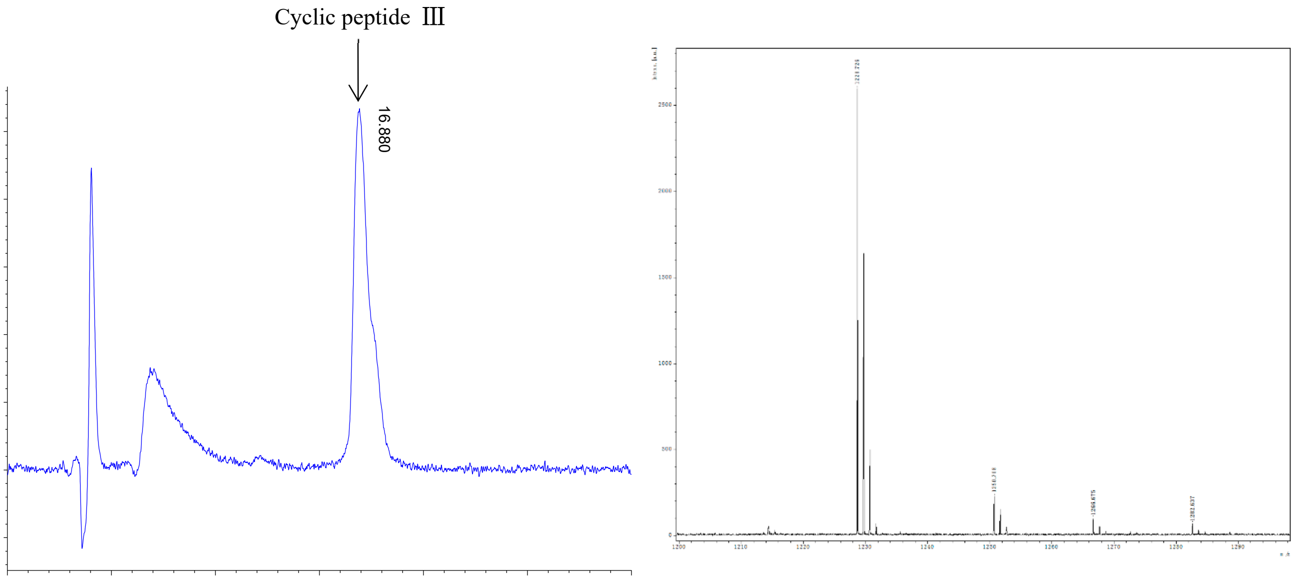

Figure S3. HPLC chromatograms and identification of cyclic peptide Ⅲ. HPLC trace (left) with 23 % CH_3CN–H_2O (0.1% TFA) as eluent system.

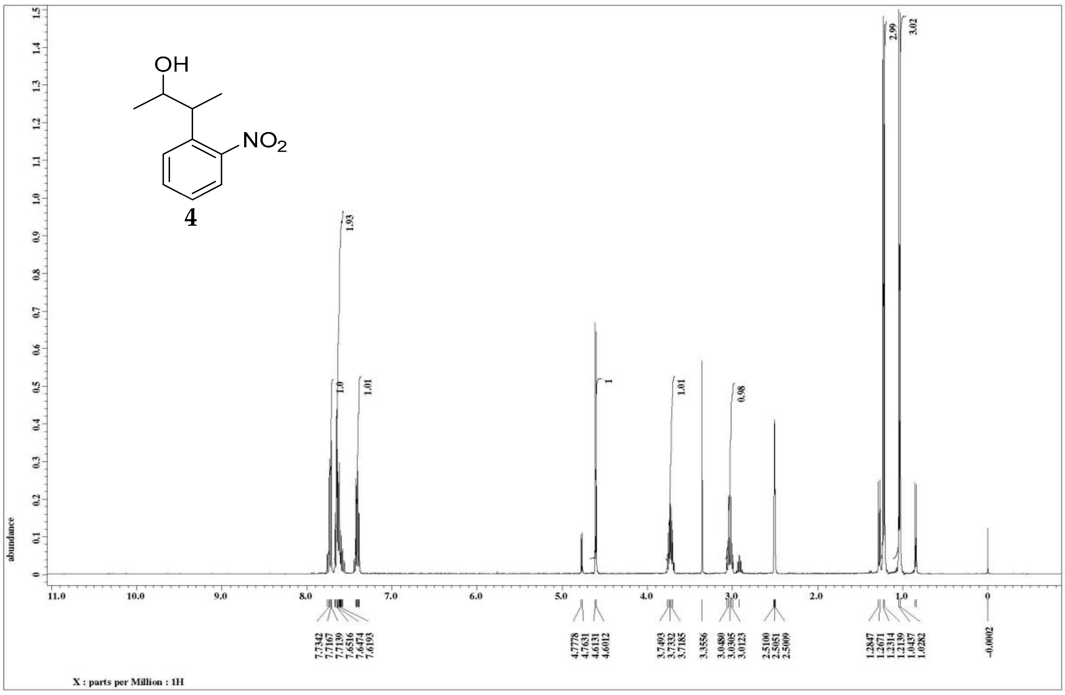

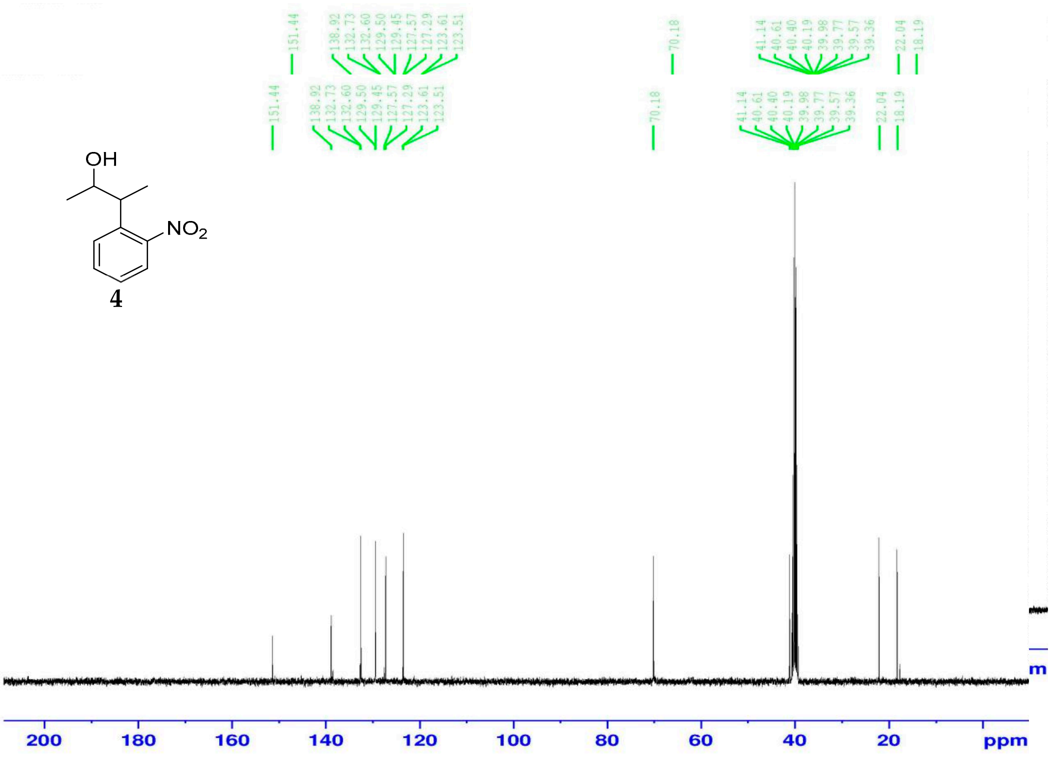

Figure S4. ^{1}H NMR and ^{13}C NMR spectra of compounds **4**.

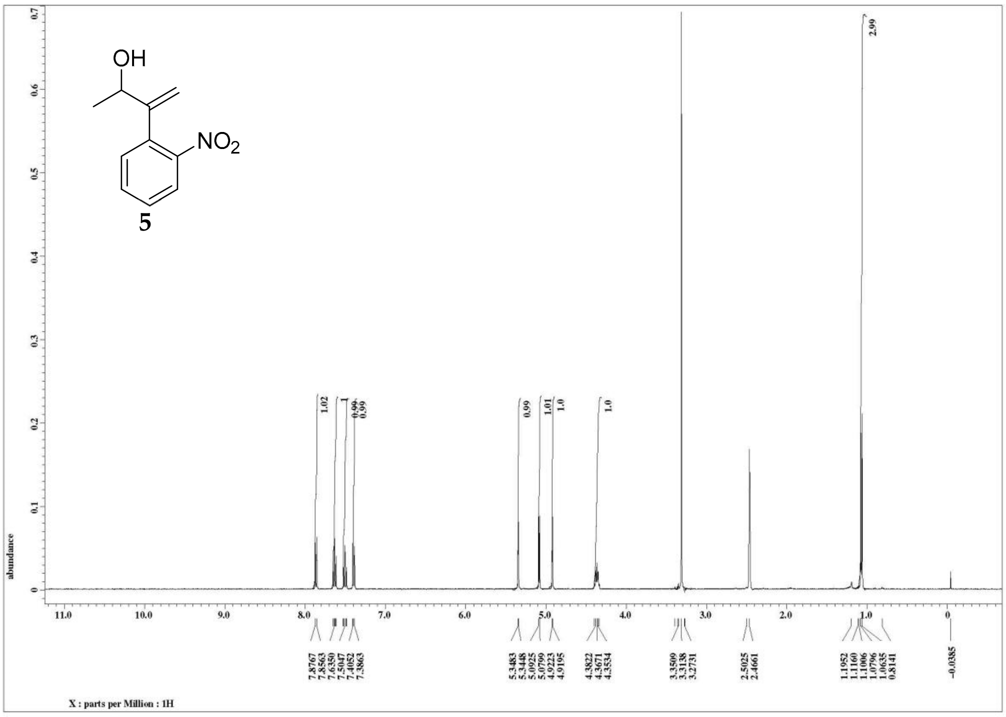

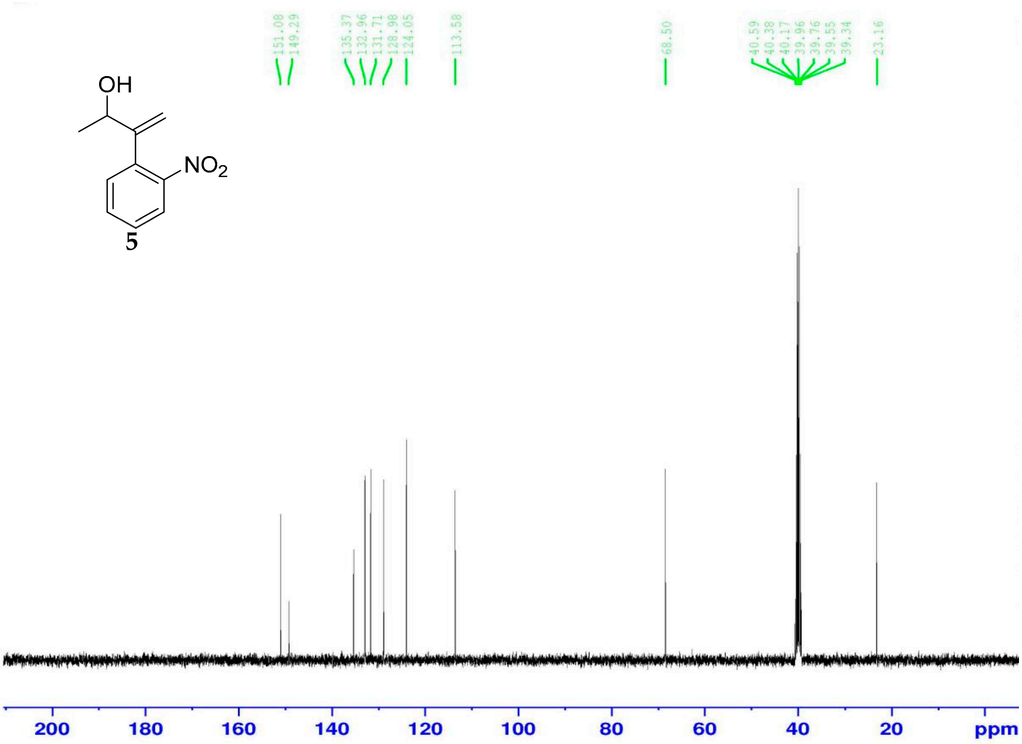

Figure S5. ^{1}H NMR and ^{13}C NMR spectra of compounds **5**.

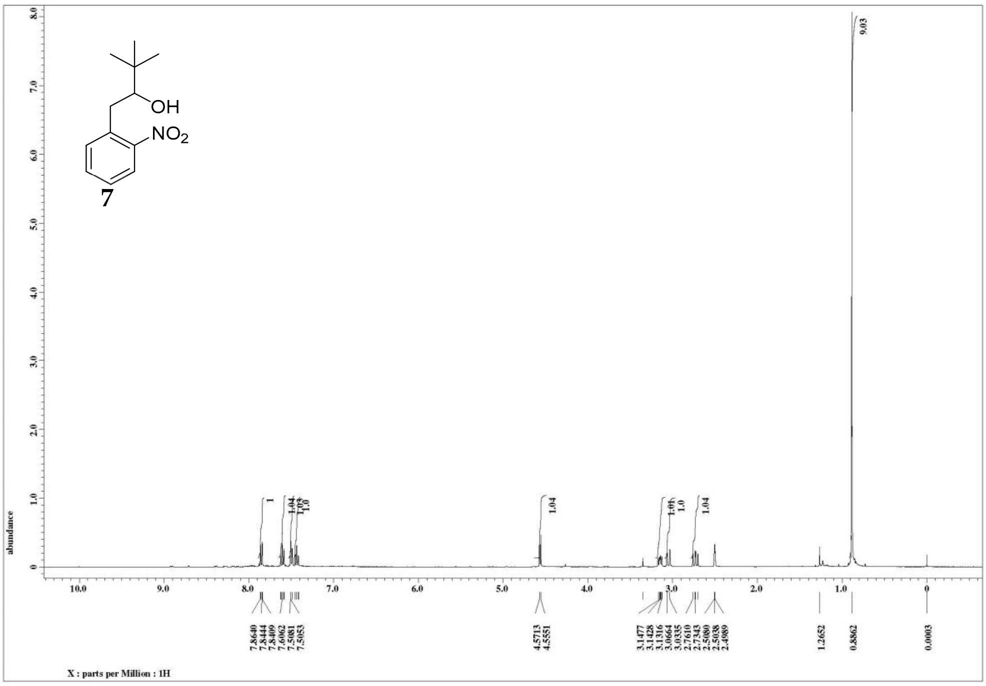

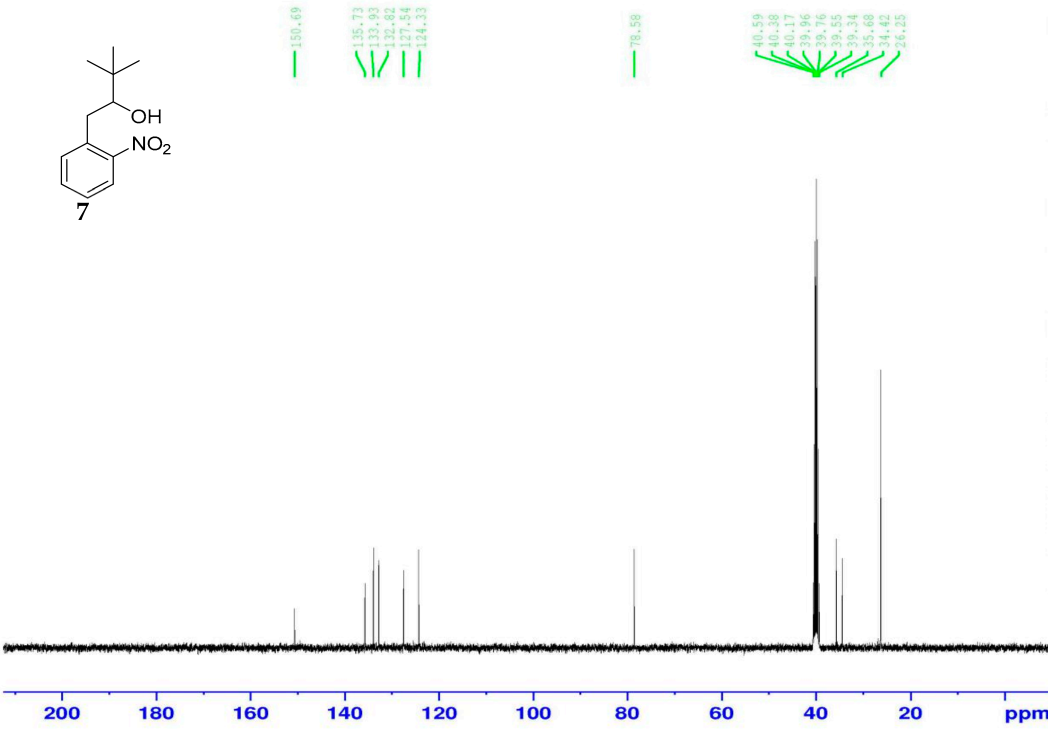

Figure S6. ^{1}H NMR and ^{13}C NMR spectra of compounds **7**.

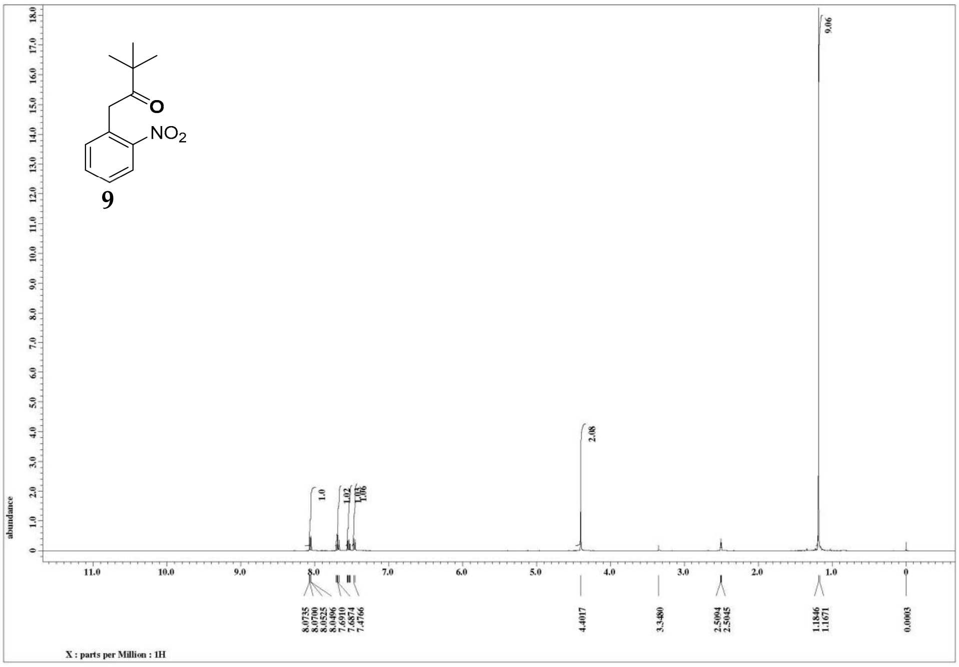

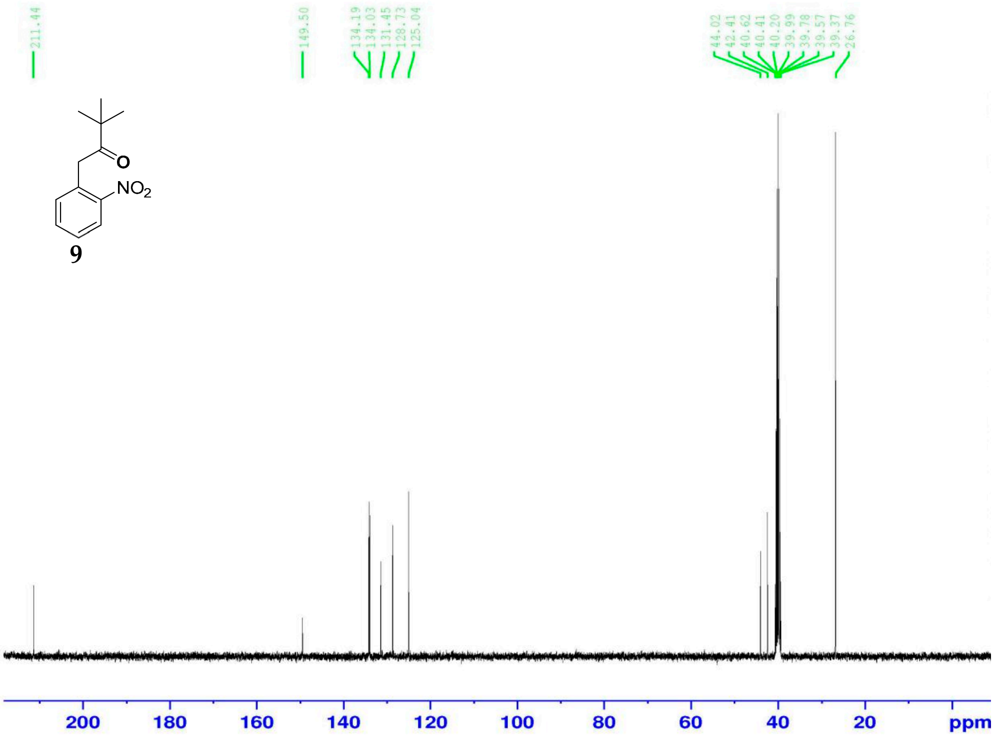

Figure S7. ^{1}H NMR and ^{13}C NMR spectra of compounds **9**.

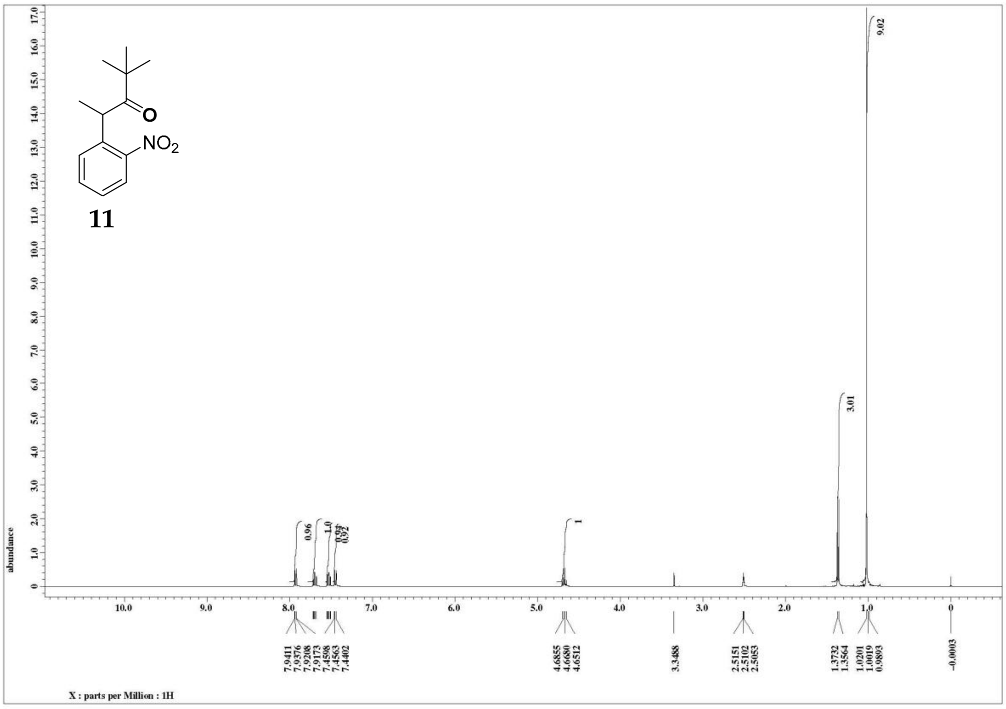

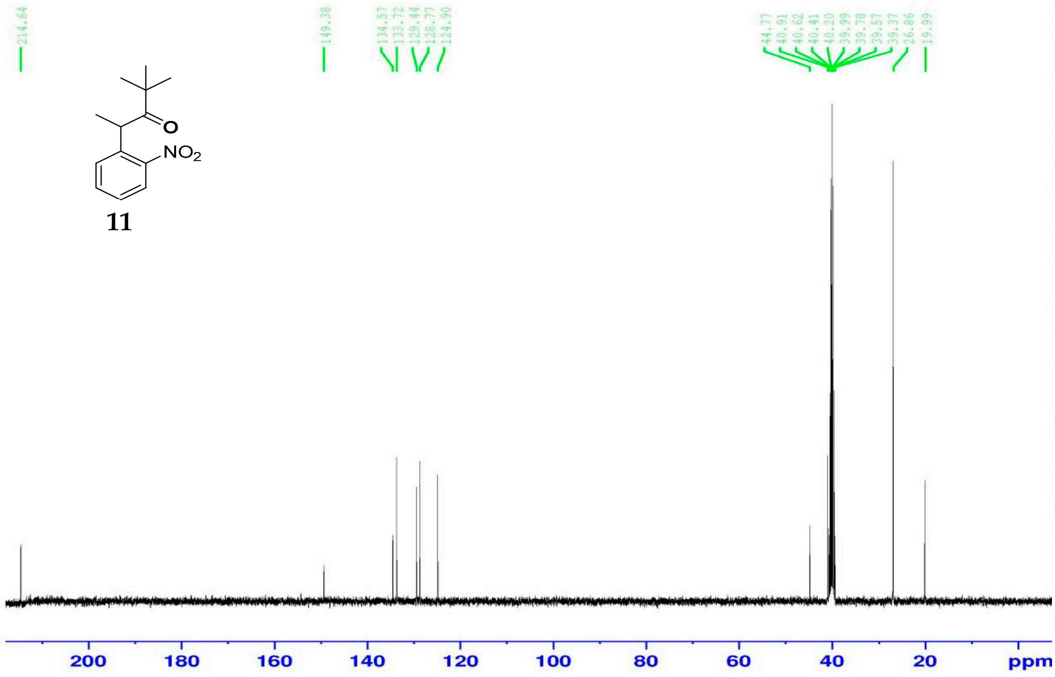

Figure S8. ^{1}H NMR and ^{13}C NMR spectra of compounds **11**.

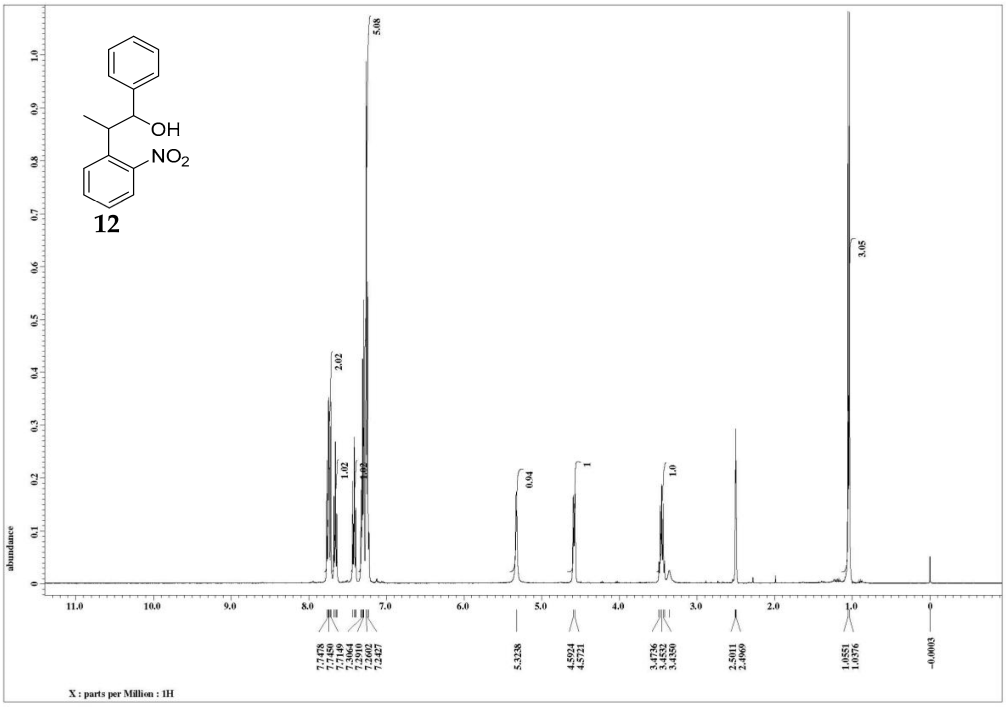

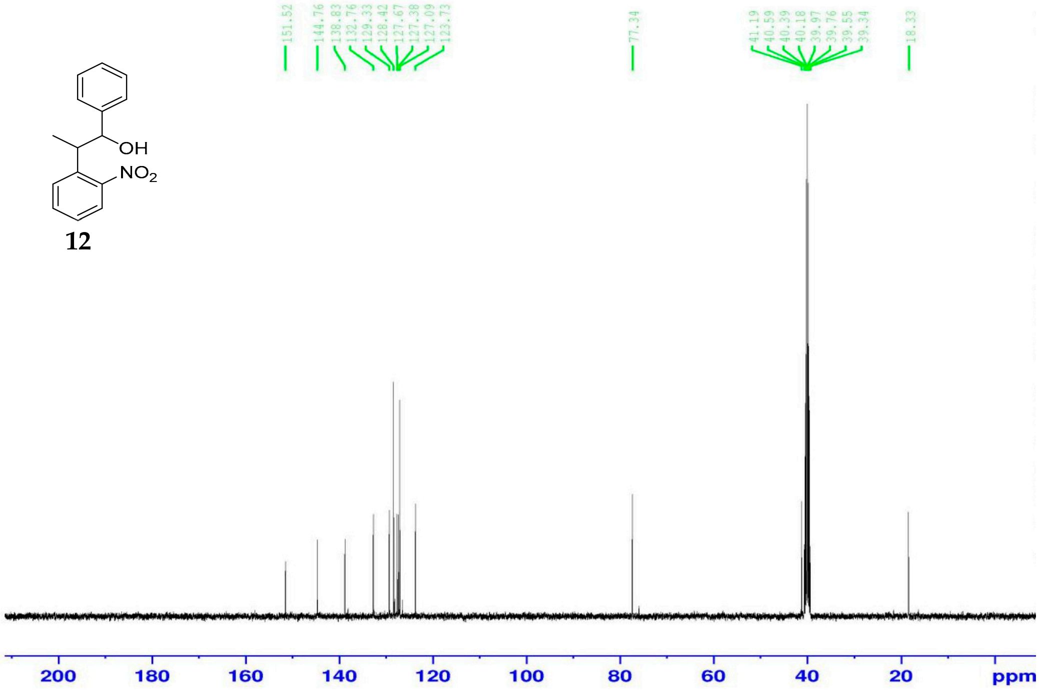

Figure S9. ^{1}H NMR and ^{13}C NMR spectra of compounds **12**.

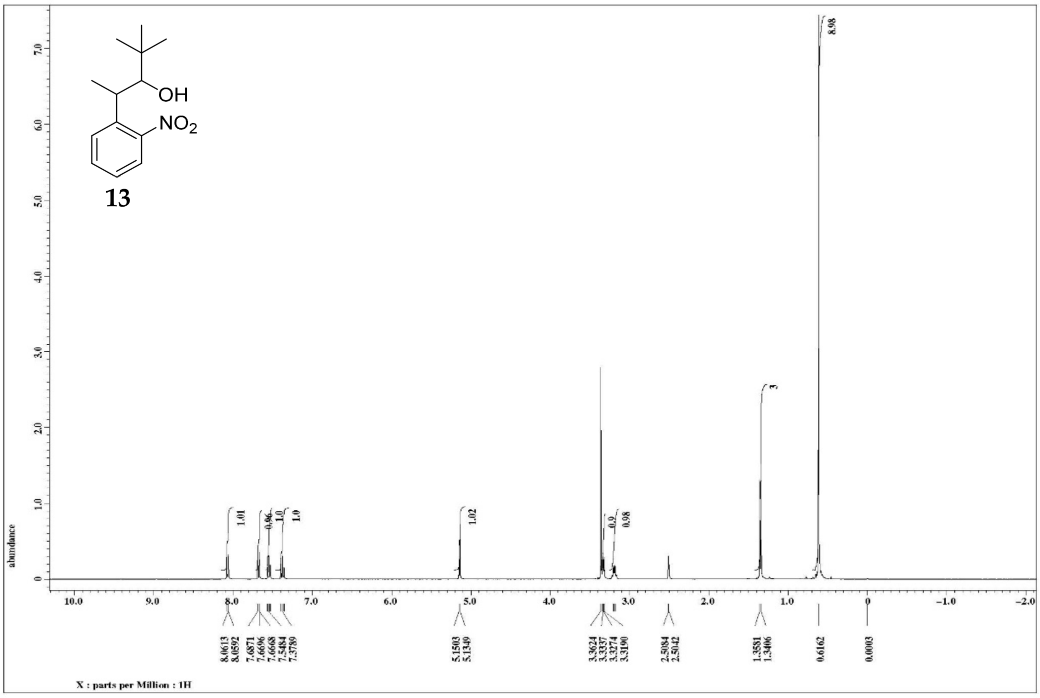

13

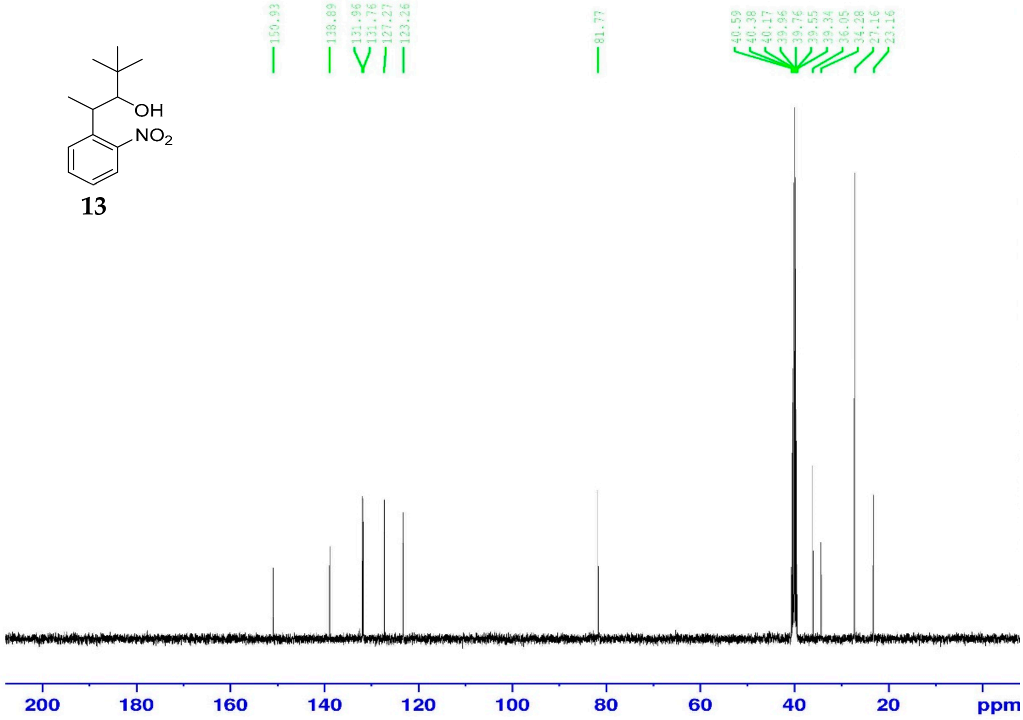

Figure S10. ^{1}H NMR and ^{13}C NMR spectra of compounds **13**.

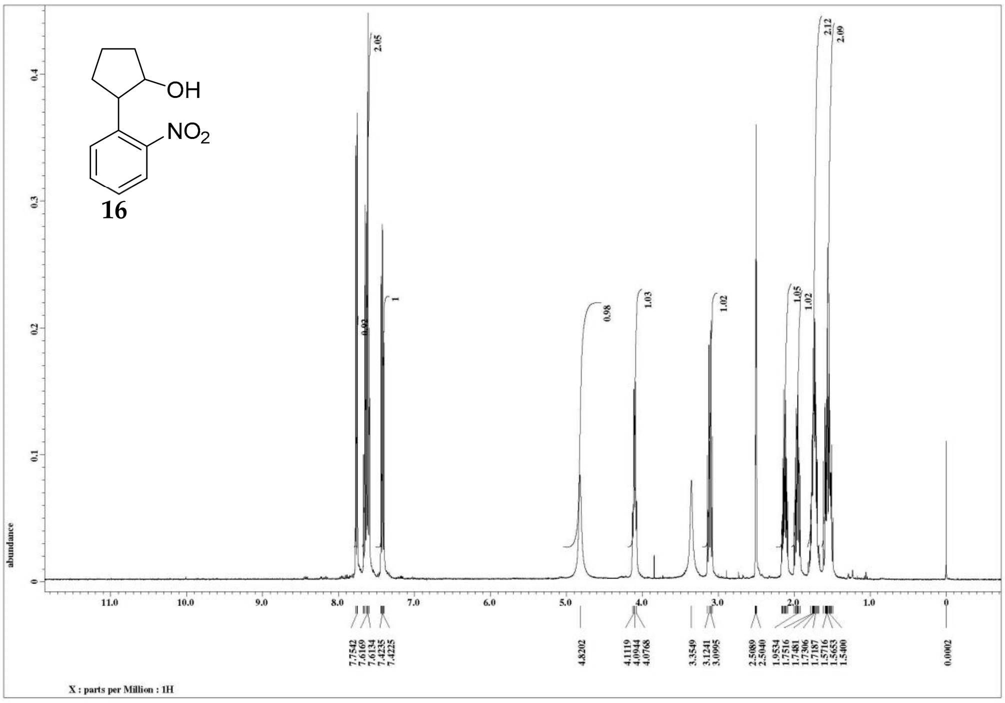

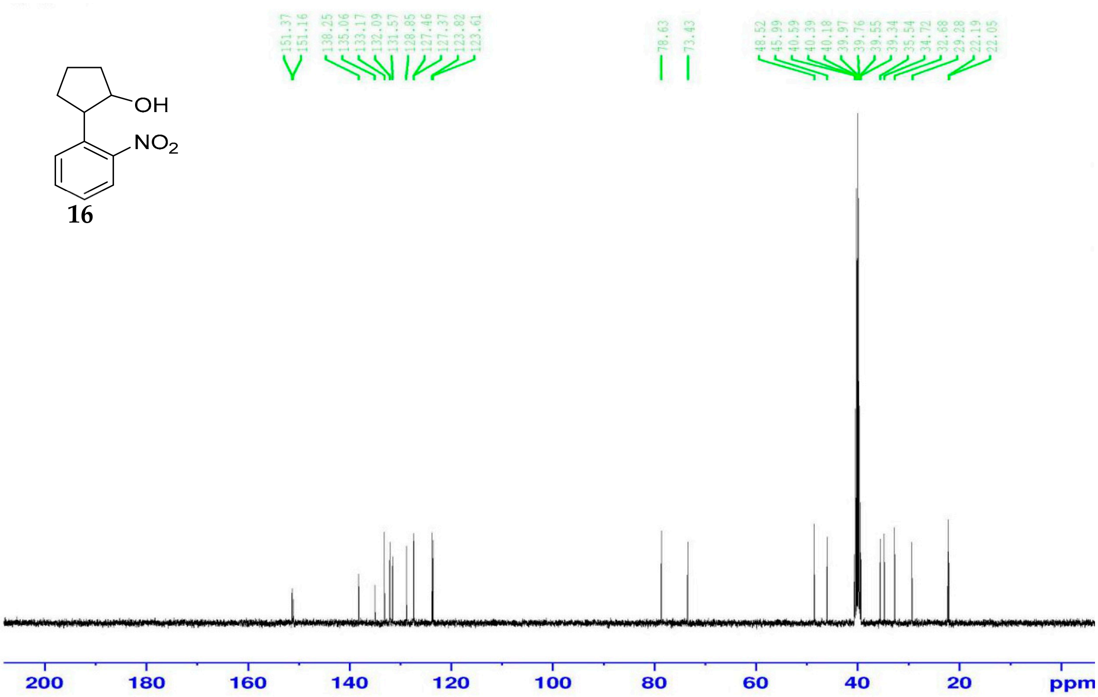

Figure S11. ^{1}H NMR and ^{13}C NMR spectra of compounds **16**.

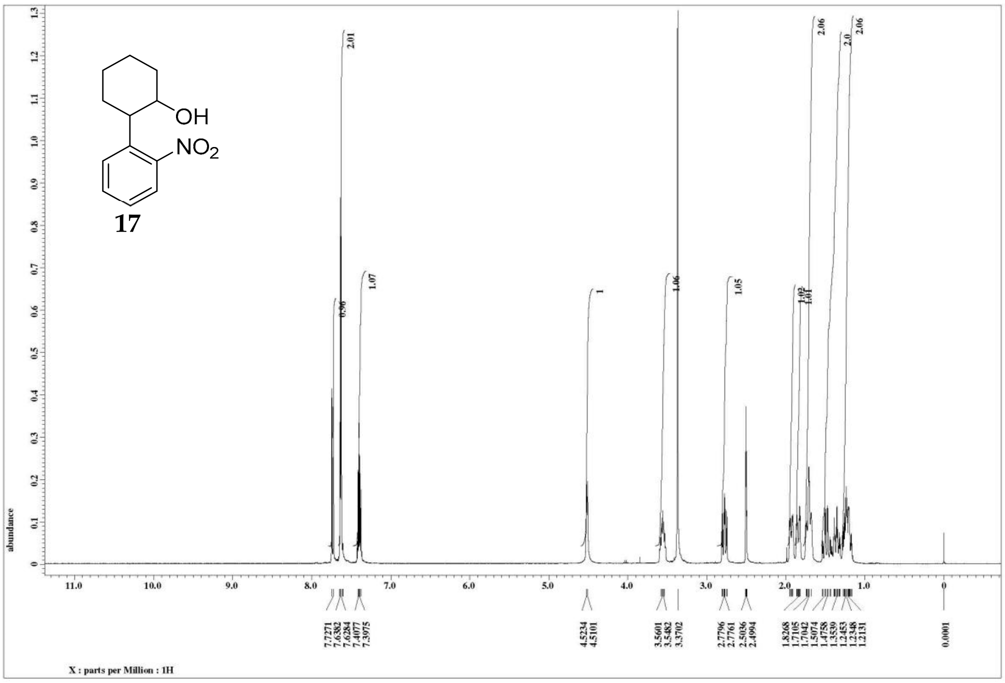

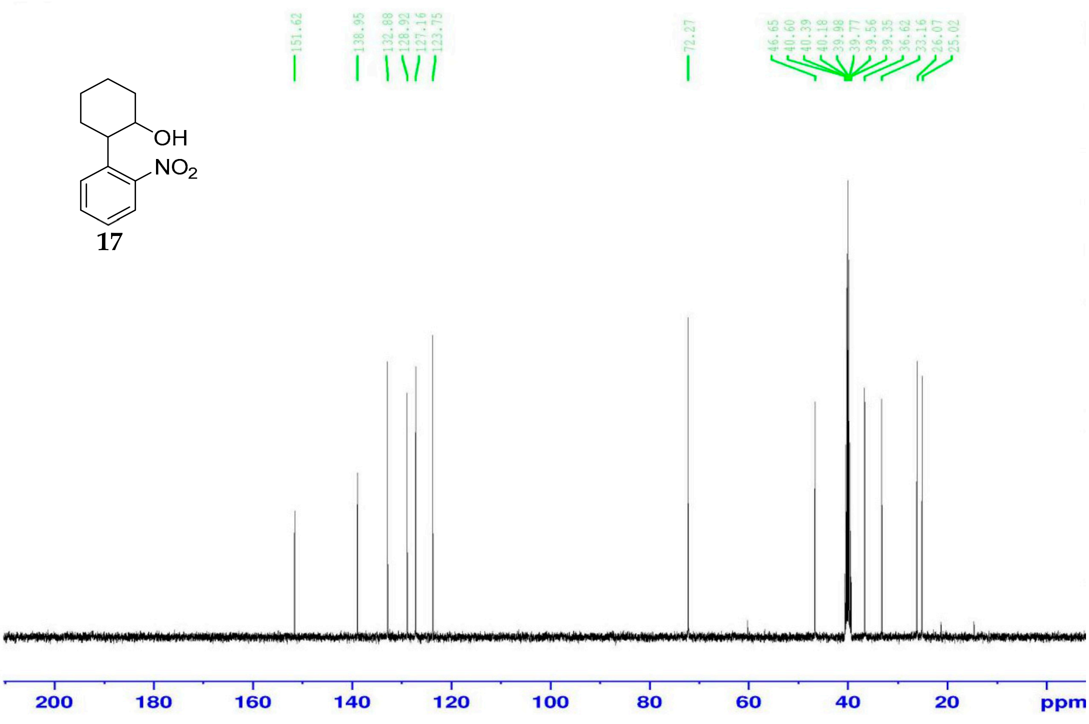

Figure S12. ^{1}H NMR and ^{13}C NMR spectra of compounds **17**.

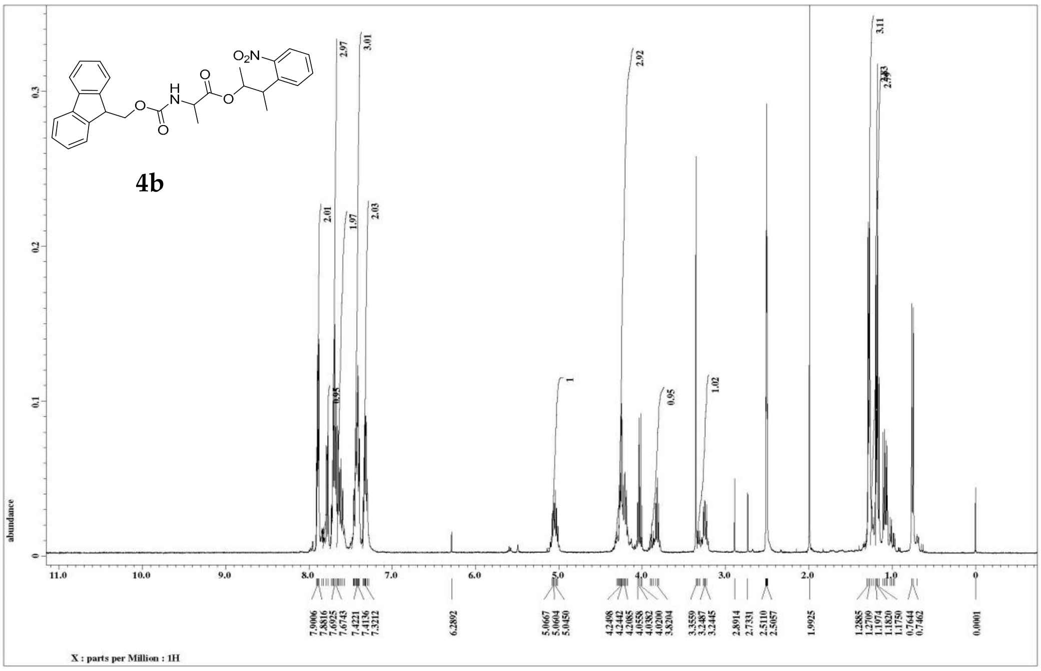

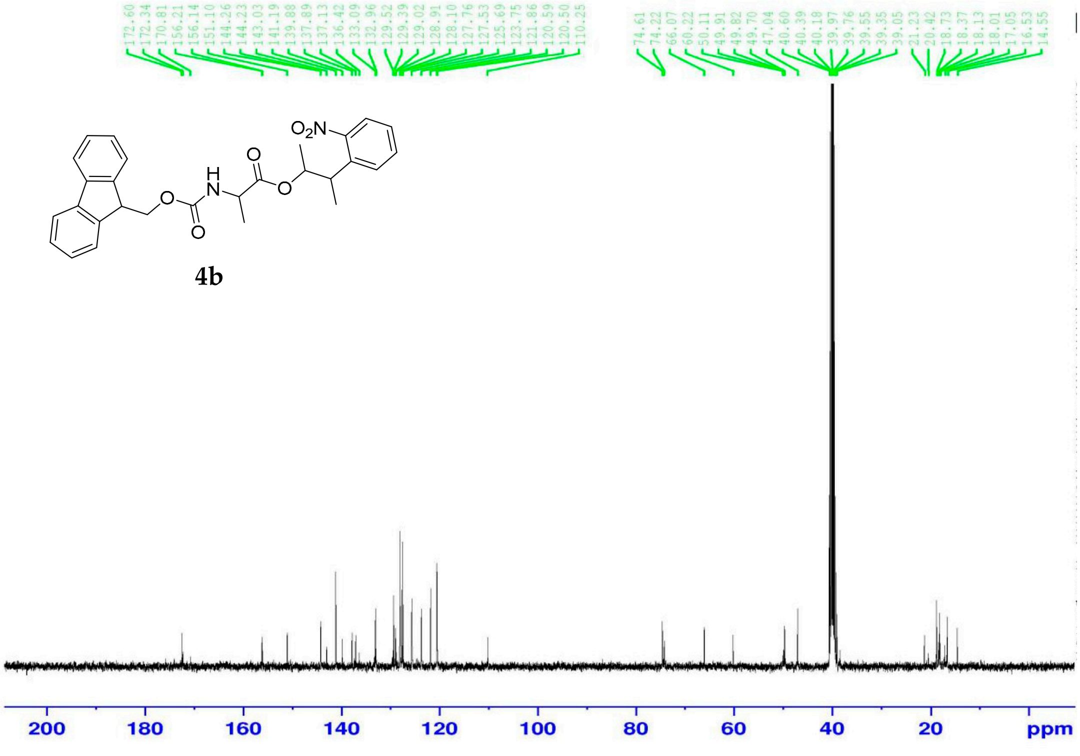

Figure S13. ^{1}H NMR and ^{13}C NMR spectra of compounds **4b**.

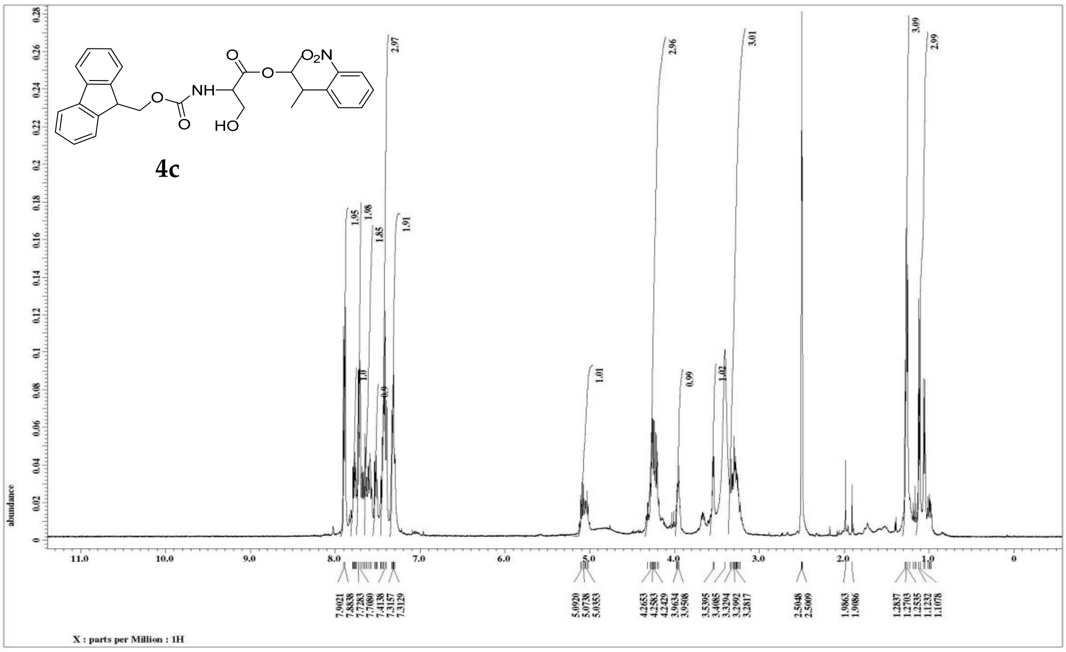

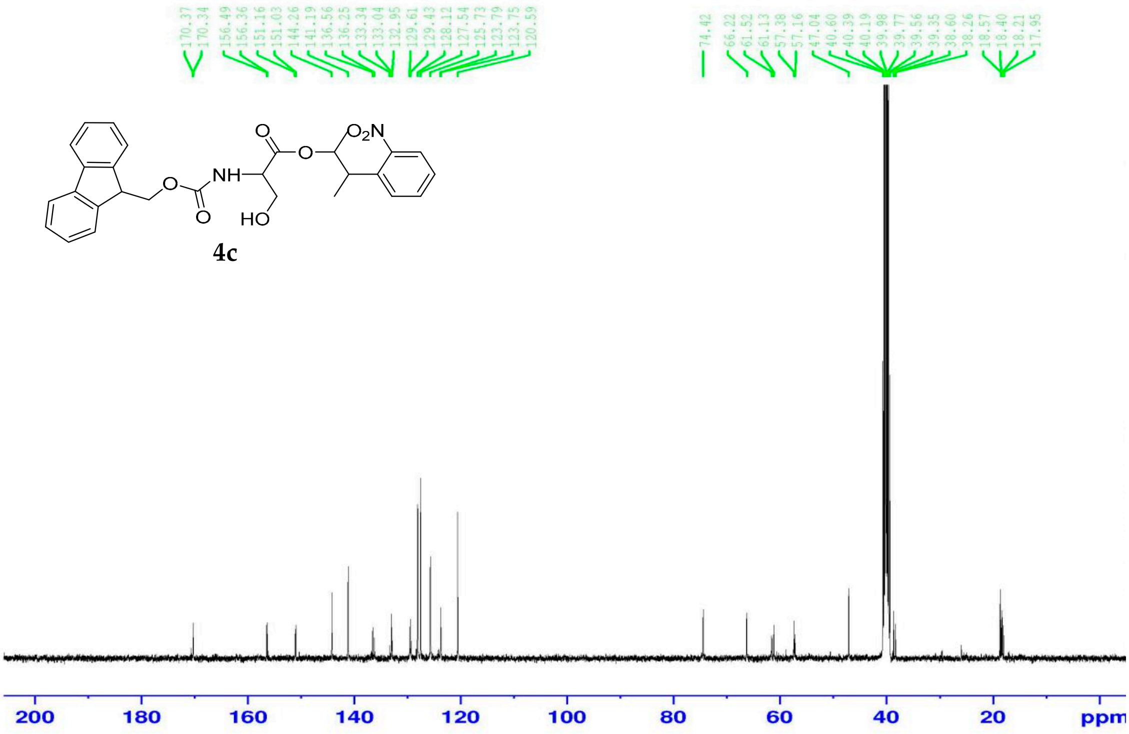

Figure S14. ^{1}H NMR and ^{13}C NMR spectra of compounds **4c**.

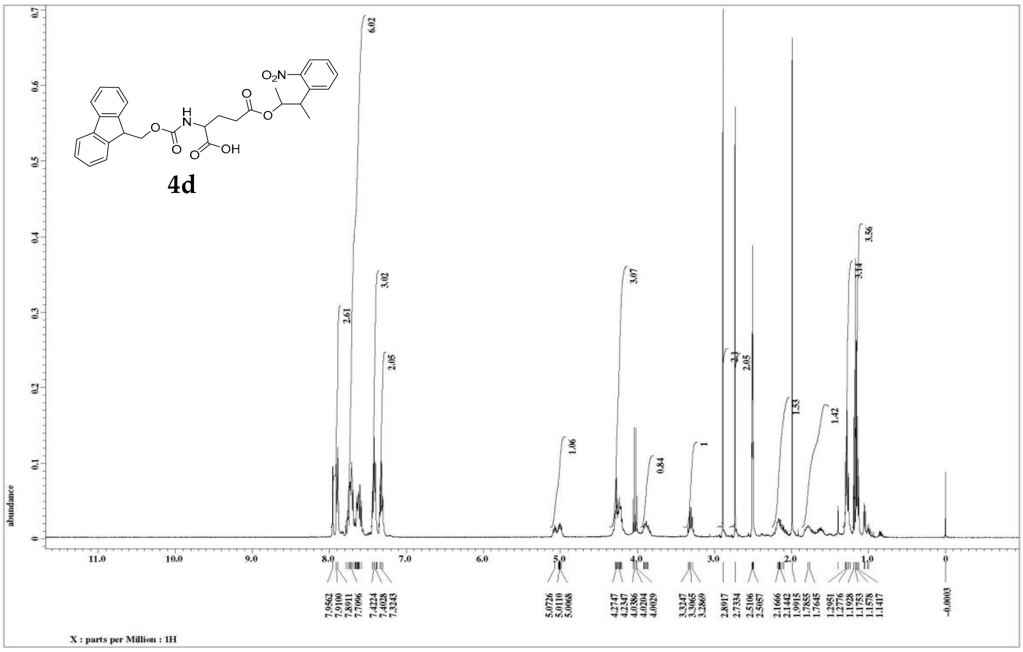

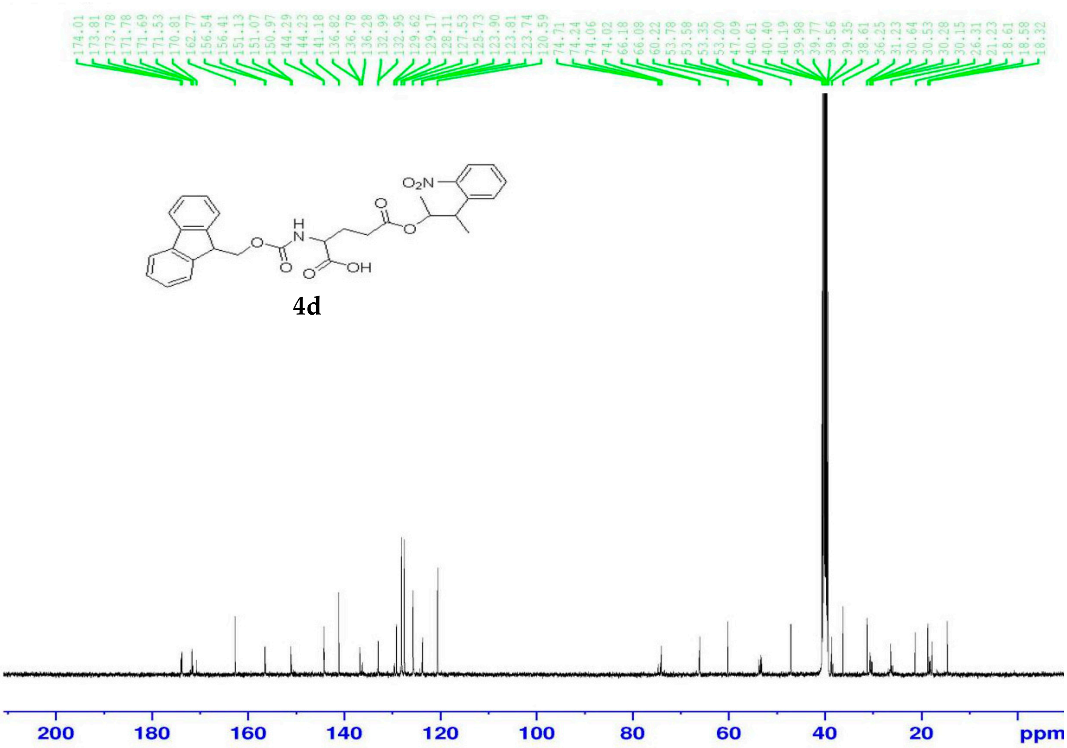

Figure S15. ^{1}H NMR and ^{13}C NMR spectra of compounds **4d**.

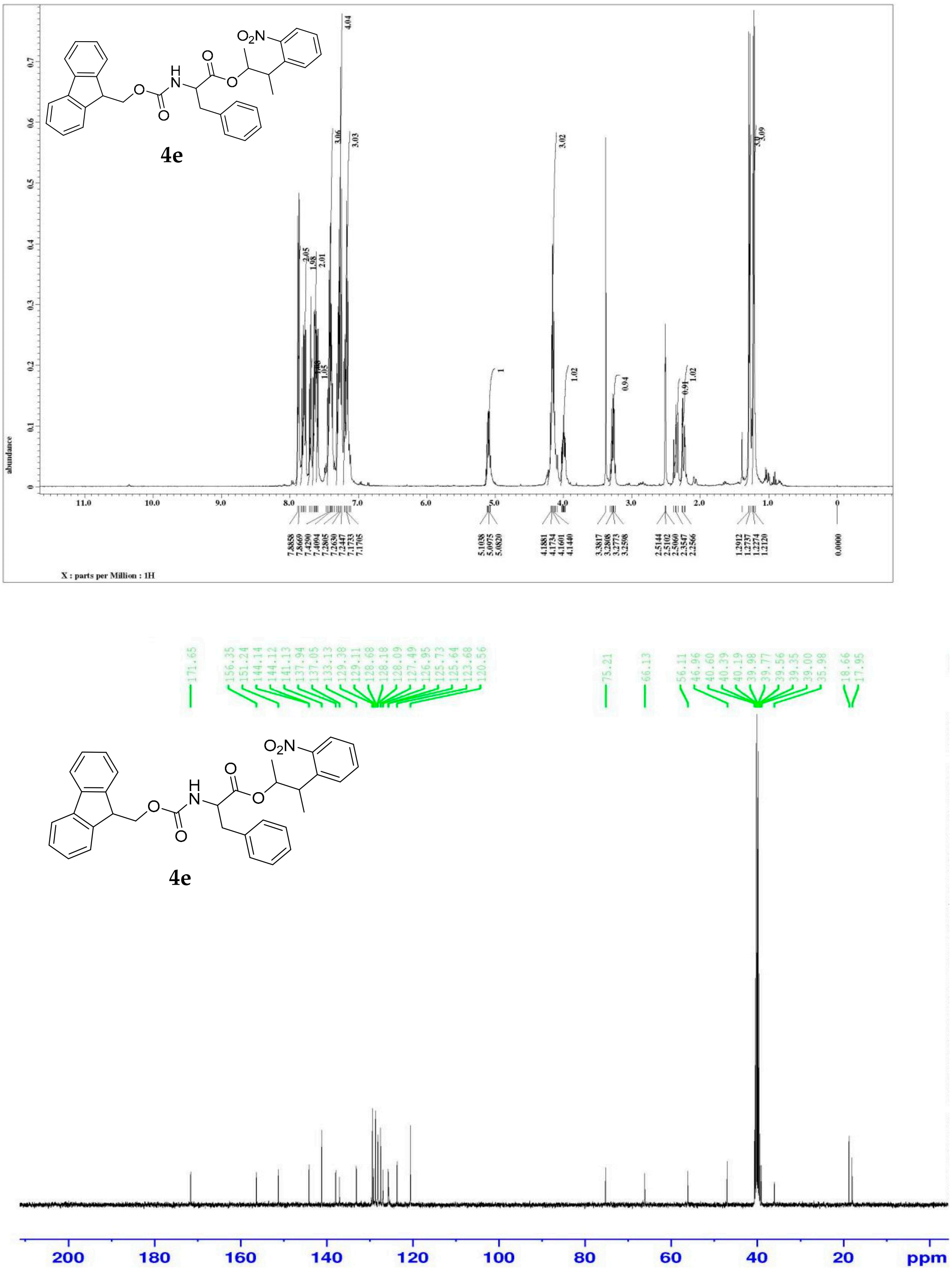

Figure S16. ^{1}H NMR and ^{13}C NMR spectra of compounds f **4e**.

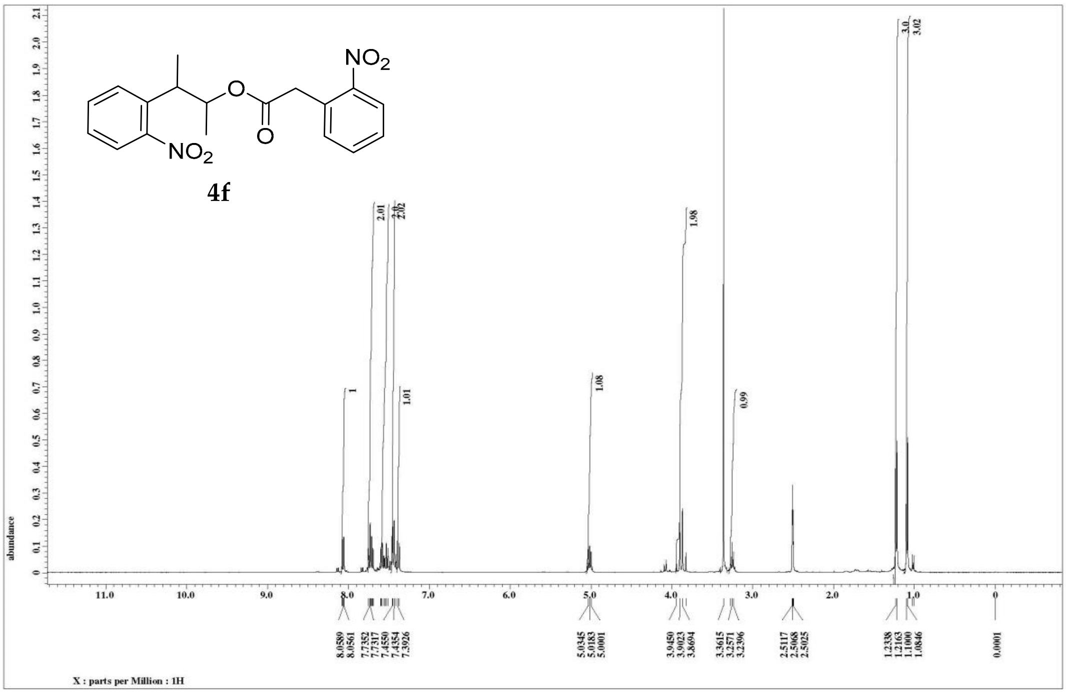

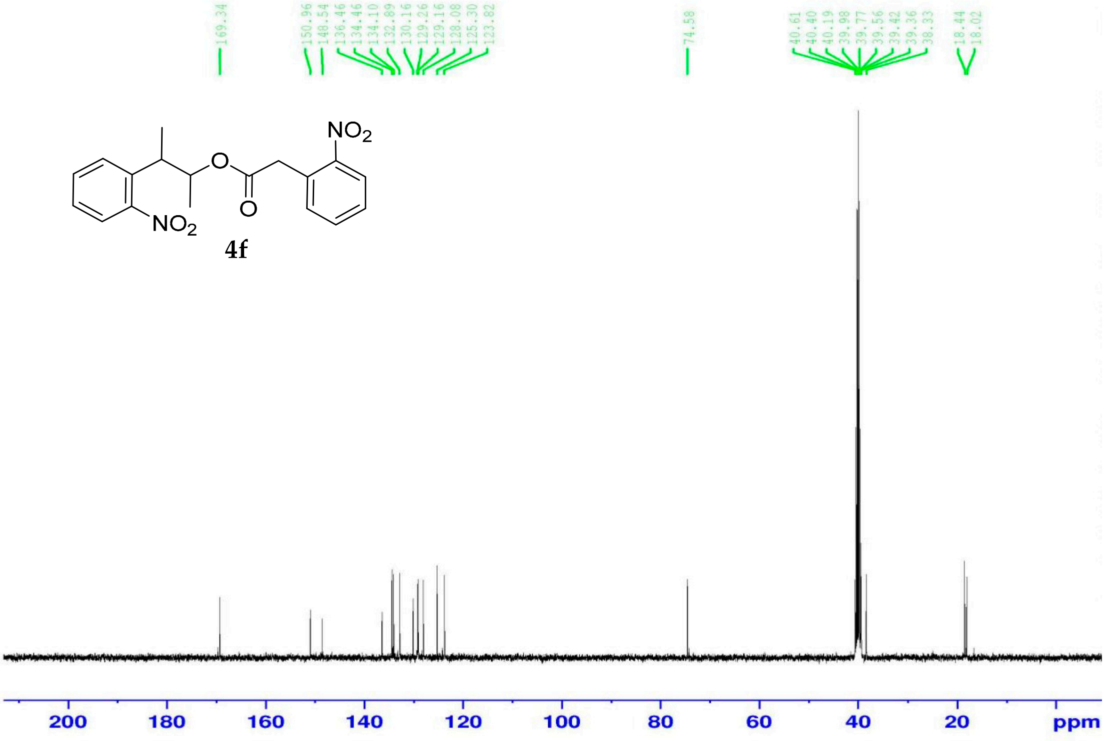

Figure S17. ^{1}H NMR and ^{13}C NMR spectra of compounds **4f**.

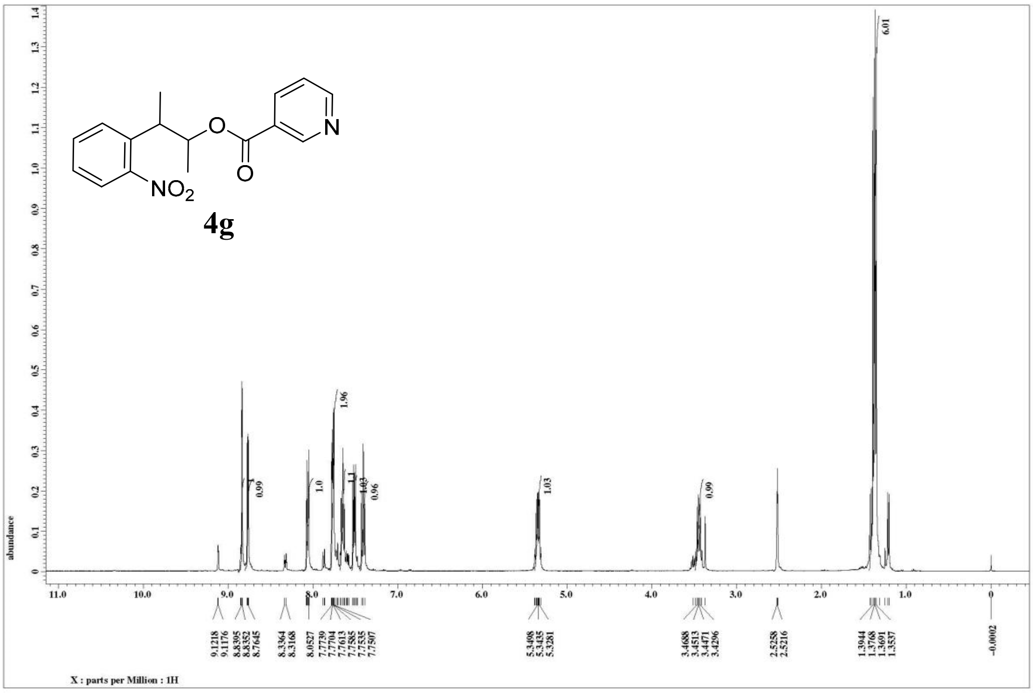

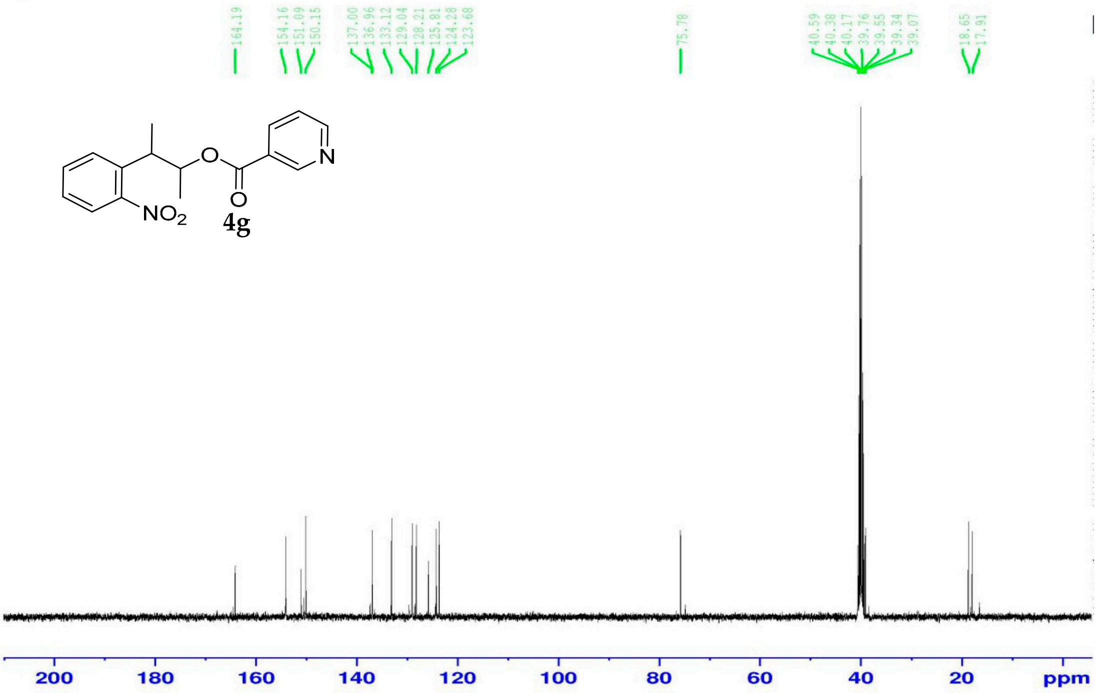

Figure S18. ^{1}H NMR and ^{13}C NMR spectra of compounds **4g**.

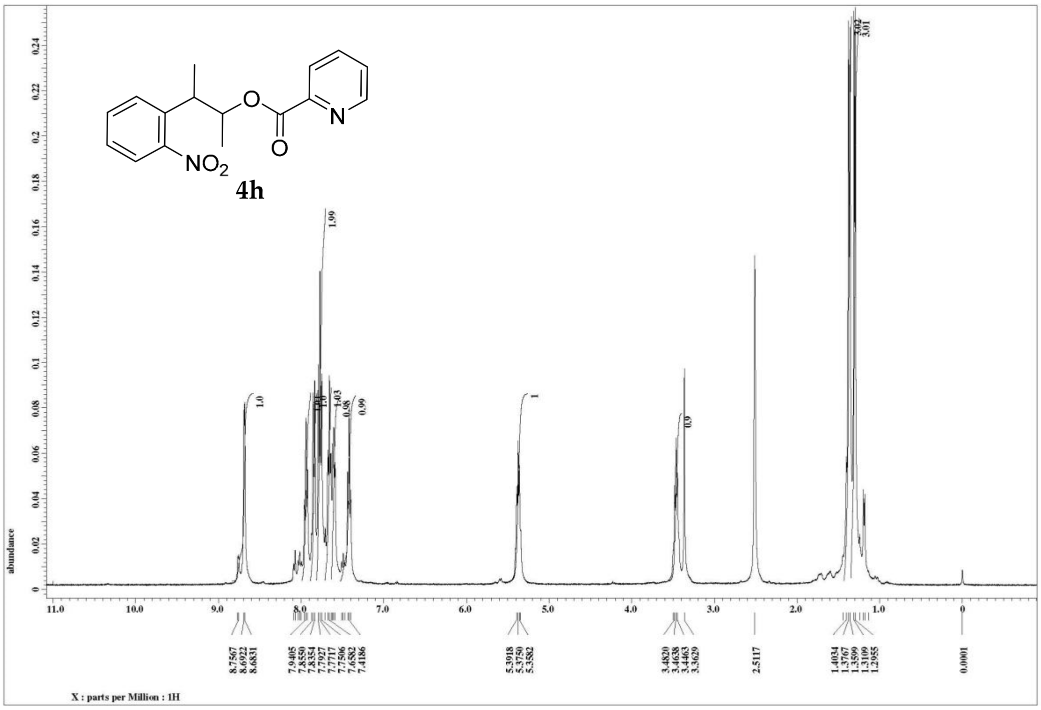

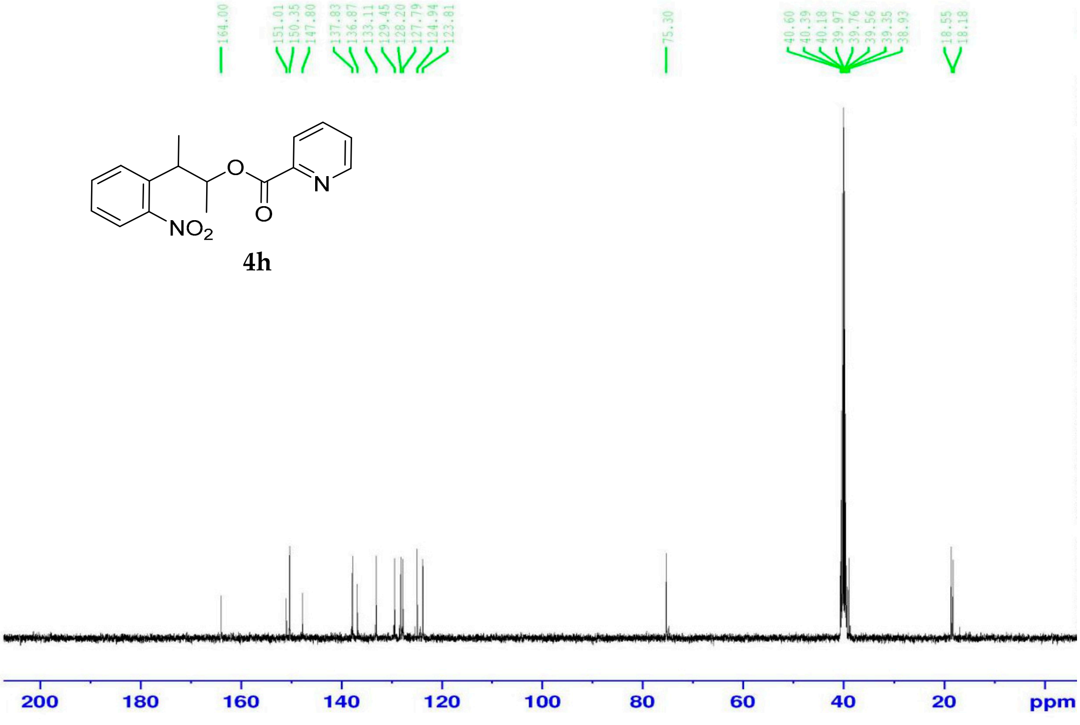

Figure S19. ^{1}H NMR and ^{13}C NMR spectra of compounds **4h.**
